# Supplementary material for: Assessing the Feasibility of a Multimodal Approach to Pain Evaluation in Early Stages after Spinal Cord Injury
Source: Int J Mol Sci. 2023 Jul 5;24(13):11122. doi: 10.3390/ijms241311122 (PMC10341965; doi:10.3390/ijms241311122)
Supplement: Supplementary file 1 [file ijms-24-11122-s001.zip › ijms-2451118-supplementary.pdf]

Supplementary Table S1 Overview of obtained data at different time points

Patient ID	Assessment	Time points			
		T1	T2	T3	T4
SWI 001	CW, QST, LEP	x	x	=T4	x
	Blood bank	m[1]	N/A	N/A	m[1]
	PSF	x	x	=T4	x
SWI 002	CW, QST	x	x	=T4	x
	Blood bank	x	N/A	N/A	x (24)
	PSF	x	x	=T4	x
SWI 003	CW, QST	m[2]	x	=T4	x
	Blood bank	x	N/A	N/A	x (30)
	PSF	x	x	=T4	x
SWI 004	CW, QST	m[2]	m[3]	m[3]	m[3]
	Blood bank	x	N/A	N/A	x (45)
	PSF	x	x	x	x
SWI 005	CW, QST	m[4]	x	=T4	x
	Blood bank	m[1]	N/A	N/A	m[1]
	PSF	x	x	=T4	x
SWI 006	CW, QST	m[2]	x	=T4	x
	Blood bank	x	N/A	N/A	x (26)
	PSF	x	x	=T4	x
SWI 007	CW, QST	m[2]	x	x	x
	Blood bank	x	N/A	N/A	x (41)
	PSF	x	x	x	x
SWI 008	CW, QST	m[2]	x	=T4	x
	Blood bank	x	N/A	N/A	x (26)
	PSF	m[6]	m[6]	=T4	m[6]
SWI 009	CW, QST	m[5]	m[5]	m[5]	m[5]
	Blood bank	x	N/A	N/A	x (38)
	PSF	x	x	m[7]	m[7]
SWI 010	CW, QST	m[5]	=T4	N/A	m[5]
	Blood bank	x	N/A	N/A	x (11)
	PSF	x	=T4	N/A	m[7]
SWI 011	CW, QST	m[5]	m[5]	=T4	m[5]
	Blood bank	x	N/A	N/A	x (23)
	PSF	x	x	=T4	x
SWI 012	CW, QST	m[5]	m[5]	=T4	m[5]
	Blood bank	x	N/A	N/A	x (24)
	PSF	x	x	=T4	x
SWI 013	CW, QST	m[5]	m[5]	N/A[8]	m[5]
	Blood bank	x	N/A	N/A	x (17)
	PSF	x	x	N/A[8]	x
SWI 014	CW, QST	m[5]	m[5]	=T4	m[5]
	Blood bank	x	N/A	N/A	x (20)
	PSF	x	x	=T4	x
SWI 015	CW, QST	m[5]	m[5]	=T4	m[5]
	Blood bank	x	N/A	N/A	x (27)
	PSF	m[7]	m[7]	=T4	x

Note: x: data obtained. m: missing data. [1]no consent for blood sample. [2]not performed, while still in ICU. [3]consent for CW/QST, but later excluded for other reasons. [4]no consent, while still in ICU. [5]no consent for CW/QST. [6]excluded from SwiSCI questionnaire for language. [7]no filled fields. [8]discharge between T2 and T3. Time point: T1 (4weeks), T2 (12 weeks), T3 (24 weeks), T4 (discharge - in bracket are indicated the weeks after injury).

Abbreviation: N/A: not applicable; ICU: intensive care unit; LEP: Laser-evoked potentials; CW: clinical workup; QST: Quantitative sensory testing; PSF: psycho-social factors.

Supplementary Figure S1 Additional pain drawings, clinical data and pain trajectories

time points	patient ID		
	**SWI 01**	**SWI 02**	**SWI 07**
**T1**	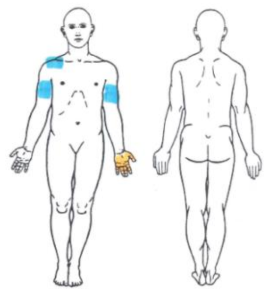	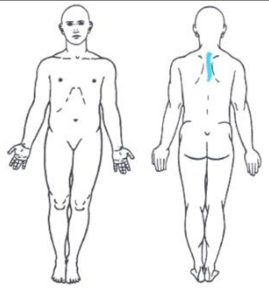	
**T2**	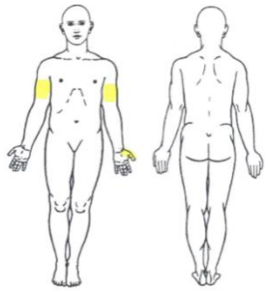	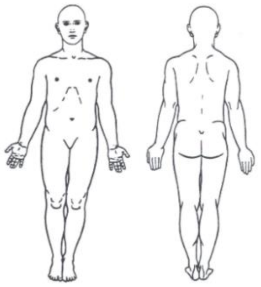	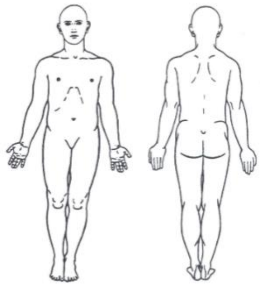
**T3**	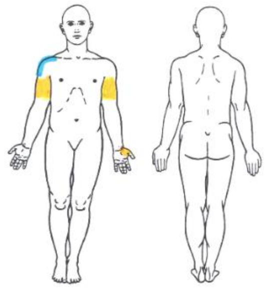	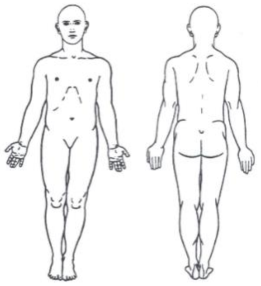	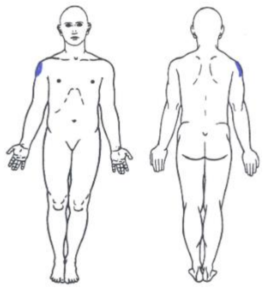
**T4**			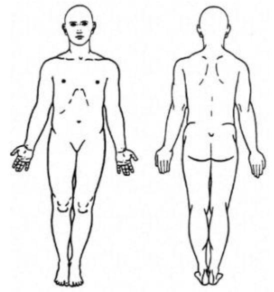
**Clinical data:**<br>NLI<br>AIS grade<br>type of palsy<br>lesion level<br>spasticity	 C3 - C1[1]<br>D<br>paraplegia<br>conus and cauda equina<br>yes	 Th4<br>A<br>paraplegia<br>spinal cord<br>yes	 C6<br>A<br>tetraplegia<br>spinal cord<br>yes
**Determination of pain trajectories at T2**	No colour shoulder right:<br>nociceptive pain resolving trajectory<br>yellow colour upper arms:<br>pain type change trajectory<br>yellow colour hand left:<br>neuropathic pain subtype change trajectory	No colour upper spine:<br>nociceptive pain resolving trajectory	
**Determination of pain trajectories at T3 (T4):**	Blue colour:<br>nociceptive pain evolving trajectory<br>Orange colour arms and hand left:<br>neuropathic pain subtype change trajectory	No colour:<br>pain free trajectory	Blue colour:<br>nociceptive pain evolving trajectory<br>*but at T4:*<br>no colour shoulder right:<br>pain free trajectory

Blue color: musculoskeletal pain. Yellow color: neuropathic at-level SCIP. Orange color: neuropathic below-level SCIP. 1: change of NLI between T2 to T3 from C3 to C1. Timepoint: T1 (4 weeks), T2 (12 weeks), T3 (24 weeks), T4 (discharge).
Abbreviation: NLI: neurological level of injury. AIS: American Spinal Injury Association (ASIA) Impairment Scale. SCIP: spinal cord injury pain.

Supplementary Figure S2 Additional QST parameters

SWI 01: QST T1-T3 **SWI 02: QST T1-T3** **SWI 07: QST T2-T4**

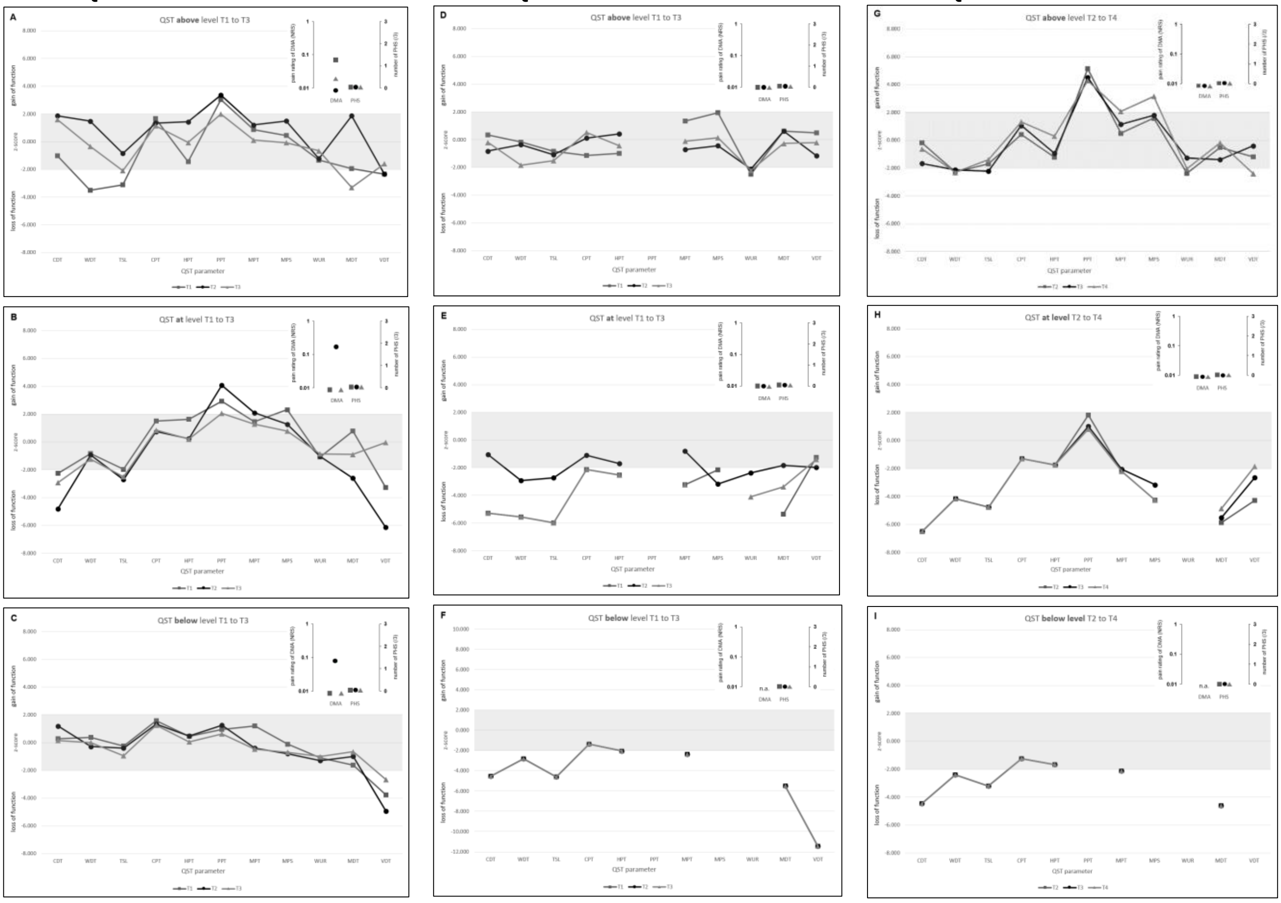

Parameters are shown in z-score values, except for PHS and DMA (inset). The grey area indicates values in normal range (-1.96 to 1.96). Z-scores below 0 indicate a loss of function in that specific QST parameter, z-scores above 0 indicate a gain of function in that specific QST parameter. Missing values are due to either deafferentation (stimuli are not felt and therefore not assessable) or spastic reactions. Time point: T1 (4 weeks), T2 (12 weeks), T3 (24 weeks), T4 (discharge).

Abbreviations: CDT: cold detection threshold; CPT: cold pain threshold; DMA: dynamic mechanical allodynia; HPT: heat pain threshold; MDT: mechanical detection threshold; MPS: mechanical pain sensitivity; MPT: mechanical pain threshold; NRS: numeric rating scale; PHS: paradoxical heat sensations; PPT: pressure pain threshold; QST: quantitative sensory testing; TSL: thermal sensory limen; VDT: vibration detection threshold; WDT: warm detection threshold; WUR: wind-up ratio.

Supplementary Table S2 Clinical and pain related characteristics of patients investigated for neurological and pain evaluation at timepoints T2 and T3

Participants		**T2**	**T3**
Sex (female, male)	N=7 (2f, 5m)		
Age (at accident), years (mean ± SD)	40.7 ± 14.9		
Neurological level of injury (NLI)	Cervical	2	2
	Thoracic	3	3
	Lumbar	2	2
ASIA Impairment scale (AIS)	A	3	2
	B	2	2
	C	-	-
	D	2	3
Lesion type	Spinal cord	5	5
	Conus and cauda equina	2	2
	Cauda equina	0	0
Type of paralysis	Paraplegia	5	5
	Tetraplegia	2	2
Spasticity	Yes	6	6
	No	1	1
Medication	Antiepileptics	3	2
	Antidepressants	2	1
	Opioids	1	2
	Analgesics	3	3
	Baclofen	1	2
	Other antispastic	0	2
Pain types and subtypes*#			
1. Nociceptive pain	Musculoskeletal pain	1	5
	Visceral pain	0	0
	Other nociceptive pain	0	0
2. Neuropathic pain	At level	2	1
	Below level	1	3
	Other neuropathic pain		
3. Other pain		0	0
4. Unknow pain		0	0
Number of different pain subtypes per patient#	One pain type	3	4
	Two pain types	0	2
	Three pain types	0	
Pain site$	Shoulder	0	2
	Upper arm	2	2
	Hand	1	1
	Back	0	3
	Chest front side	2	4
	Thigh	1	2
Pain descriptions per pain type*£ (nociceptive/neuropathic pain)	1. Hot-burning	0/0	3/0
	2. Tingling	0/2	0/2
	3. Pricking	1/3	4/2
	4. Pins and needles	0/1	2/1
	5. Sharp	1/2	1/1
	6. Shooting	0/1	1/0
	7. Squeezing	0/1	0/0
	8. Painful cold	0/1	0/1
	9. Electric shock-like	0/2	2/2
	Others (dragging, pressure-like)	0/0	2/0
Mean pain intensity, NRS per pain subtype (mean ± SD)	Musculoskeletal pain	(4.0 ± 0.0)	(4.2 ± 2.7)
	At-level SCIP	(3.5 ± 0.5)	(5.0 ± 0.0)
	Below-level SCIP	(5.0 ± 0.0)	(3.7 ± 1.2)

Note: *According to Bryce *et al*. 2012. #bilateral occurrence of pain subtypes in the same dermatomal distribution was counted as one pain type. $in bilateral occurrence of pain sites, each side was counted. £multiple announcement possible. Time point: T2 (12 weeks), T3 (24 weeks).
Abbreviations: AIS: ASIA (American Spinal Injury Association) Impairment Scale; NRS: numerical rating scale; SCIP: spinal cord injury pain.

Supplementary Table S3 Overview of QST-parameters per patient, which could not be collected or interpretation is limited due to technical reasons

**Patient ID Time point**	**AIS**	**Paralysis type**	**QST**		
			Above level	At level	Below level
SWI 01					
T2	D	tetraplegic	nil	nil	nil
T3	D	tetraplegic	nil	nil	nil
SWI 02					
T2	A	paraplegic	PPT[2]	PPT[2]	CDT[3], WDT[4], TSL[4], CPT[3], HPT[4], PPT[2], MPT[4,6], MPS[4,6], WUR[4], MDT[4,6], VDT[3], PHS[8], DMA[5]
T3	A	paraplegic	PPT[2]	CDT[3], WDT[4], TSL[4], CPT[3], HPT[4], PPT[2], MPT[4,6], MPS[4,6]	CDT[3], WDT[4], TSL[4], CPT[3], HPT[4], PPT[2], MPT[4,6], MPS[4,6], WUR[4], MDT[4,6], VDT[3], PHS[8], DMA[5]
SWI 03					
T2	D	paraplegic	nil	CPT[3], HPT[4], PPT[2], WUR[5]	CDT[3], PPT[5], WUR[6]
T3	D	paraplegic	nil	PPT[2], MPT[6], WUR[6]	PPT[6], WUR[6]
SWI 05					
T2	B	paraplegic	PPT[1]	CPT[3], HPT[4], PPT[1], WUR[4]	HPT[4], PPT[1], MPT[4], WUR[4]
T3	B	paraplegic	PPT[1]	HPT[4], PPT[1], MPT[4], WUR[4], DMA[5]	HPT[4], PPT[1], MPT[4], WUR[4,6], DMA[5]
SWI 06					
T2	D	paraplegic	nil	nil	CDT[3], WDT[4], TSL[4], CPT[3], HPT[4], PPT[2,5], MPT[4], MPS[5], WUR[5], MDT[4], PHS[8], DMA[5]
T3	C	paraplegic	nil	identical to Below level QST[7]	CDT[3], WDT[4], TSL[4], CPT[3], HPT[4], PPT[2,5], MPT[4], WUR[5], MDT[4]
SWI 07					
T2	A	tetraplegic	nil	CDT[3], WDT[4], TSL[4], CPT[3], HPT[4], MPT[4], WUR[5]	CDT[3], WDT[4], TSL[4], CPT[3], HPT[4], PPT[5,6], MPT[4], MPS[5], WUR[5], MDT[4], VDT[3], PHS[8], DMA[5]
T3	A	tetraplegic	nil	CDT[3], WDT[4], TSL[4], CPT[3], HPT[4], WUR[5]	CDT[3], WDT[4], TSL[4], CPT[3], HPT[4], PPT[5,6], MPT[4], MPS[5], WUR[5], MDT[4], VDT[3], PHS[8], DMA[5]
SWI 08					
T2	A	paraplegic	nil	PPT[1] (Th3)	CDT[3], WDT[4], TSL[4], CPT[3], HPT[4], PPT[1], MPT[4], MPS[5], WUR[5], MDT[4], VDT[5], PHS[8], DMA[5]
T3	A	paraplegic	nil	PPT[1] (Th3)	CDT[3], WDT[4], TSL[4], CPT[3], HPT[4], PPT[1], MPT[4], MPS[5], WUR[5], MDT[4], VDT[5], PHS[8], DMA[5]

Note: [1] parameter not testable in thoracic area, risk of rib fracture due to supposed osteoporosis
[2] parameter not testable in atrophied muscles and risk of injury due to too much pressure
[3] minimal value is entered, but patient didn't felt stimulus due to deafferentation
[4] maximal value is entered, but patient didn't felt stimulus due to deafferentation
[5] parameter not determined due to the deafferentation (not possible to perform due to not feeling any stimulus)
[6] parameter not testable due to spasticity
[7] for clinical reason At-level dermatome is identical with below level dermatome, therefore not tested
[8] parameter not assessable, due to non-perceiving temperature

Abbreviations: AIS: ASIA (American Spinal Injury Association) Impairment Scale; CDT: cold detection threshold; CPT: cold pain threshold; DMA: dynamic mechanical allodynia; HPT: heat pain threshold; MPS: mechanical pain sensitivity; MDT: mechanical detection threshold; MPT: mechanical pain threshold; PHS: paradoxical heat sensations; PPT: pressure pain threshold; QST: quantitative sensory testing; TSL: thermal sensory limen; VDT: vibration detection threshold; WDT: warm detection threshold; WUR: wind-up ratio.

Supplementary Table S4 Standard Operating Procedure for QST, adapted for use in SCI for single parameters

Temperature testings	The temperature margins of QST are defined per protocol to be 0°C and 50°C. Hence, values displayed by the device outside this range (>50°C or <0°C, due to technical variations) will be documented as 50°C or 0°C respectively.<br><br>In case that patient completely fails to perceive stimuli due to deafferentation, the minimum and maximum values are entered into the datasheet. as 0°C/50°C for CDT and WDT. In this case, for PHS, n.a. is entered in the data sheet.<br><br>In patients that do not perceive warmth (WDT) nor cold (CDT) and which can't discriminate between these two sensations (TSL), CPT and HPT is evaluated during the TSL measurement by asking for a burning, stabbing, drilling or dragging sensation with the objective to prevent overstraining of the skin in paraplegic patients. This is necessary because the temperature has already reached the maximum of 50°C and the minimum of 0°C six times each.
PPT	To avoid the risk of skin damage, a maximum pressure of $8kg/cm^2$ was defined. If this maximum pressure was not perceived due to deafferentation or can't be measured due to muscular atrophy, n.p. is entered in the data sheet.
MPT/MDT	In case that patient completely fails to perceive mechanical stimuli due to deafferentation the maximum value is entered into the datasheet as 724.08mN for MDT and MPT.
MPS/DMA	In patients who rate the pinprick stimulus with 0, normally the correction value 0.1 is substituted to calculate the z-score (which is a logarithmic value). This might lead to a normal z-score in young patients. Therefore, a correction value of 0.01 was used.<br><br>In patients who do not perceive pinprick stimuli due to deafferentation, n.a. is entered into the datasheet.<br><br>In patients who do not perceive cotton wool stimuli, Q-tip stimuli and brush stimuli due to deafferentation, n.a. is entered into the datasheet.
WUR	In WUR prinprick type is not defined, depending on MPS-rating of each individual. If possible, always use the same pinprick, e.g. neck 128mN, thenar 64mN, foot 128mN.<br><br>Pinpricks should be selected as such, that the rating of the single stimulus is above 0 in at least 3 out of 5 stimulations and below 10, to ensure a pathological value<br><br>If the WUR is not testable due to deafferentation or spasticity, n.p. is noted.

Note: Table shows adapted handling of several QST-parameters in addition to Rolke et al., Pain, 2006. n.p.: not performed. n.a.: not applicable.

Supplementary Table S5 Standard Operating Procedure for QST, adapted for use in SCI to define areas for measurement vibration detection threshold and pressure pain thresholds

**Dermatome (ASIA)**	**Vibration detection threshold (VDT)**	**Pressure pain threshold (PPT)**
Face	Processus zygomaticus	M. masseter
C2	not testable, use the face areas	not testable, use the face areas
C3	Medial clavicula	M. trapezius, near the neck
C4	Acromion	M. trapezius, lateral
C5	Epicondylus lateralis	M. biceps, ventral
C6	Thumb basal joint	M. abductor pollicis brevis
C7	Os Metacarpale III, distal end	M. triceps
C8	Processus styloideus ulnae	M. abductor digiti minimi
Th1	Epicondylus medialis	M. abductor digiti minimi
Th2-T7	Rib in projection of the corresponding dermatome	Not testable (according ASIA there is no defined muscle) Additionally, there is a risk of rib fractures due to osteoporotic conditions in SCI patients
Th8-10	Rib in projection of the corresponding dermatome, in the area of the middle axillary line (lateral thorax)	Not testable (according ASIA there is no defined muscle) Additionally, there is a risk of rib fractures due to osteoporotic conditions in SCI patients
Th11-12	most probably not testable, potentially testable on a "free rib" or alternatively on vertebral body Th11/Th12	Not testable (according ASIA there is no defined muscle) Additionally, there is a risk of rib fractures due to osteoporotic conditions in SCI patients
L1	Vertebral body L1	Ventral upper thigh, dermatome L1
L2	Iliac crest on the side	Ventral upper thigh, dermatome L2
L3	Dorsal iliac crest, in the middle, 5cm caudal	Ventral upper thigh, dermatome L3
L4	Condylus medialis femoris (inner knee)	Vastus medialis above the knee
L5 Foot	Malleolus medialis	M. abductor hallucis
S1	Malleolus lateralis	M. gastrocnemius, caput laterale
S2	Tuber ischiadicum	M. biceps femoris

Note: C2-C8: cervical dermatomes. Th1-TH12: thoracic dermatomes. L1-L5: lumbar dermatomes. S1-S2: sacral dermatomes.

Supplementary Table S6 Clinical data characteristics of blood biomarkers analysis participants (SwiSCI questionnaire)

Characteristics of participants		**T1**	**T4**
Sex (female, male)	n=13 (1f, 12m)		
Age (at accident), years (mean ± SD)	47.7 ± 17.6		
Neurological level of injury (NLI)	Cervical	3	3
	Thoracic	5	4
	Lumbar	5	6
ASIA Impairment scale (AIS)	A	8	6
	B	-	-
	C	2	-
	D	3	5
Type of paralysis	Paraplegia	10	10
	Tetraplegia	3	3
Medication	Anxiolytic - Antidepressant	9	6
	Antithrombotic	13	7
	Opioids	5	2
	Non-opioid Analgesics	8	2

Note: Time point: T1 (4 weeks), T4 (discharge).

SupplementaryTable S7 Inflammation and pain related blood biomarkers

	CTR	T1	T4
**Gene expression** (Relative mRNA expression)			
MCP-1	2.0±0.4 (1.6-2.3)	1.0±0.6 (0.7-1.3)	1.0±0.3 (0.8-1.1)
IL-6	6.4±2.5 (4.4-8.4)	2.5±1.9 (1.5-3.6)	4.2±2.4 (2.8-5.5)
IL-10	0.3±0.2 (0.1-0.5)	0.6±0.3 (0.4-0.7)	0.3±0.1 (0.3-0.4)
CCL3	3.6±1.2 (2.6-4.6)	3.3±1.7 (2.3-4.2)	4.5±1.6 (3.6-5.4)
BDNF2	1.6±0.8 (0.9-2.2)	4.7±4.1 (2.5-7)	4.5±5.4 (1.6-7.4)
COX2	1.0±0.2 (0.8-1.1)	0.9±0.5 (0.6-1.2)	0.9±0.2 (0.7-1.0)
FKBP5	1.2±0.7 (0.6-1.7)	1.2±0.6 (0.9-1.5)	1.2±0.3 (1.1-1.4)
GM-CSF	1.8±0.7 (1.2-2.4)	1.1±0.7 (0.7-1.5)	1.4±0.6 (1.1-1.7)
IL-1 beta	1.3±0.6 (0.9-1.8)	1.0±0.5 (0.7-1.3)	0.9±0.4 (0.7-1.1)
IL-2	2.4±1.5 (1.2-3.6)	1.7±0.6 (1.3-2.0)	2.0±1.5 (1.2-2.9)
IL-4	1.0±0.7 (0.4-1.5)	0.7±0.4 (0.5-0.9)	0.9±0.6 (0.5-1.2)
IL-8	2.2±1.9 (0.7-3.7)	1.1±0.7 (0.7-1.5)	1.8±1.2 (1.2-2.4)
TNF-alpha	2.5±0.6 (2.0-3.0)	3.7±2.8 (2.1-5.2)	3.5±1.6 (2.6-4.4)
VEGFA	1.2±0.5 (0.8-1.6)	1.0±0.4 (0.7-1.2)	1.0±0.2 (0.8-1.1)
**Immunoassay**			
CRP (mg/L)	5.0±5.3 (0-10.2)	11.6±7.8 (7.4-15.8)	6.0±4.2 (3.7-8.3)
Calprotectin (mg/mL)	2.6±1.5 (1.1-4.1)	7.5±10.2 (1.9-13.0)	2.1±1.1 (1.6-2.7)
OPN (ng/mL)	28.7±9.4 (19.5-38)	72.2±29.1 (56.4-88)	58.5±23 (46-70.9)
ICAM-1 (ng/mL)	4.2±0.9 (3.3-5.1)	4.1±1.6 (3.2-5.0)	5.6±3.2 (3.9-7.3)
Cystatin C (ng/mL)	275±59 (217-333)	380±67 (344-417)	447±119 (382-512)
IL-1 beta (pg/mL)	66±6 (59-72)	102±84 (50-154)	85±43 (55-115)
IFN-alpha2 (pg/mL)	31±19 (9-52)	48±69 (0-99)	41±64 (0-83)
IFN-gamma (pg/mL)	13	49±69 (0-109)	52±77 (0-119)
TNF-alpha (pg/mL)	44±34 (0-91)	102±139 (0-238)	75±129 (0-178)
MCP-1 (pg/mL)	463±244 (224-703)	432±171 (339-524)	385±185 (285-486)
IL-6 (pg/mL)	ND	77±44 (34-120)	83±47 (30-136)
IL-8 (pg/mL)	ND	79±45 (29-130)	85±30 (55-114)
IL-10 (pg/mL)	14±11 (0-30)	44±83 (0-98)	54±103 (0-131)
IL-12p70 (pg/mL)	8	35±37 (0-71)	44±47 (0-98)
IL-17A (pg/mL)	2.9±0.8 (2.0-3.8)	7.4±2.6 (3.8-10.9)	7.2±4.4 (2.3-12.2)
IL-18 (pg/mL)	162±69 (95-230)	193±135 (119-266)	160±120 (95-225)
IL-23 (pg/mL)	8.7±7.6 (1.3-16.2)	31.9±71.7 (0-74.3)	52.9±107 (0-127)
IL-33 (pg/mL)	36.6±8.9 (27-47)	96.2±92.6 (28-165)	87.5±94 (18-157)
Myoglobin (ng/mL)	74±29 (45-102)	145±176 (49-240)	114±103 (59-170)
NGAL (ng/mL)	342±46 (297-387)	528±328 (349-706)	532±322 (357-707)
MMP-2 (ng/mL)	215±41 (175-256)	204±33 (186-222)	224±41 (202-246)
MPO (ng/mL)	157±17 (140-173)	203±107 (145-261)	205±122 (138-271)
SAA (mg/mL)	1.1±1.7 (0-2.8)	1.6±1.6 (0.7-2.5)	0.8±0.9 (0.3-1.2)
IGFBP-4 (ng/mL)	185±58 (128-242)	201±42 (178-224)	240±80 (197-284)
VCAM-1 (ng/mL)	305±89 (218-392)	292±93 (242-343)	322±80 (279-366)
MMP-9 (mg/mL)	1.2±0.3 (0.9-1.5)	1.4±0.8 (1.0-1.9)	1.4±0.7 (1.1-1.8)

Time point: T1 (4 weeks), T4 (discharge). CTR: healthy able-bodies controls.
Note: values are shown as mean ± SD (95%CI). ND= not detectable.

SupplementaryTable S8 QST-feedback questionnaire

Statement	**T1**	**T2**	**T3/T4**
	n=2	*n=4*	*n=5*
1) The **benefit** of the measurement was explained to me in an understandable manner	4.0 ± 0.0	4.0 ± 0.0	3.8 ± 0.4
2) The **course of action** and the procedures were explained to me in an understandable manner	4.0 ± 0.0	4.0 ± 0.0	4.0 ± 0.0
3) I was well **supervised**	4.0 ± 0.0	4.0 ± 0.0	4.0 ± 0.0
4) The measurement was well **organized**	3.0 ± 0.0	3.5 ± 0.0	3.6 ± 0.5
5) The **duration** of the measurement was too long for me	1.0 ± 0.0	0.75 ± 0.4	1.4 ± 1.0
6) The measurement had **negative consequences** for me	0.5 ± 0.5	0.25 ± 0.4	0.4 ± 0.5
7) I would **recommend participation** in this research project to equally affected individuals	3.0 ± 0.0	3.5 ± 0.0	3.4 ± 0.5
8) In case of negative impact, please describe separately	none	none	none
9) Use separate space for further comments	none	none	"Sometimes it is very difficult to explain why the sensitivity is higher on some days and lower on others."

Note: Statements 1 - 7 (mean ± SD) were rated with according to the following scale:
4 = "fully applies", 3 = "predominantly applies", 2 = "partly applies", 1= "rather does not apply", 0 = "not at all".
Questions 8 and 9 where open questions, only one patient responded to question 9.
Time point: T1 (4 weeks), T2 (12 weeks), T3 (24 weeks), T4 (discharge).
Abbreviations: QST: quantitative sensory testing.

Supplementary Table S9 Primer sequences used in gene expression assay

Target	Sequence Forward	Sequence Reverse	Calculated efficiency
TNF-alpha	cagcctcttctccttcctgat	gccagagggctgattagaga	112.0%
IL-10	tgccttcagcagagtgaaga	gcttggcaacccaggtaa	98.0%
IL-6	gatgagtacaaaagtcctgatcca	ctgcagccactggttctgt	93.3%
IL-1 beta	ctgtcctgcgtgttgaaaga	ttgggtaatttttgggatctaca	91.6%
IL-8 (1)	cctgatttctgcagctctgtg	ccagacagagctctcttccat	93.6%
IL-2	aagttttacatgcccaagaagg	aagtgaaagtttttgctttgagcta	91.5%
IL-4	gacatctttgctgcctcca	caggcagcgagtgtcctt	98.5%
COX2/PTGS2	tgggaagccttctctaacctc	ttgaatcaggaagctgctttt	83.8%
VEGF-A	gcagcttgagttaaacgaacg	ggttcccgaaaccctgag	123.0%
CCL2/MCP-1	tcaaactgaagctcgcactct	gtgactggggcattgattg	93.9%
FKBP5	acaatgaagaaagccccaca	caccattccccactcttttg	97.6%
GM-CSF	tctcagaaatgtttgacctcca	gcccttgagcttggtgag	91.9%
BDNF2	gagccagaatcggaaccac	tctcacctggtggaactcg	109.0%
CCL3	ttccgtcacctgctcagaat	tggctgctcgtctcaaagta	94.9%
PPIB	cccagttcttcatcacgaca	gtcttggtgctctccacctt	97.0%
B2M (2)	gtatgcctgccgtgtgaac	aaagcaagcaagcagaatttgg	100.0%
GAPDH	agccacatcgctcagacac	gcccaatacgaccaaatcc	88.1%

Note: Primers were designed by Roche universal Probe Library, except for:
(1) Blast Primer designing tool - NCBI
(2) Michael L. Ridley et al. DOI: 10.4049/jimmunol.1901283
